# Will future doctors know enough about stress urinary incontinence to provide proper preventive measures and treatment?

**DOI:** 10.1080/10872981.2019.1685635

**Published:** 2019-10-30

**Authors:** Joanna Witkoś, Magdalena Hartman-Petrycka

**Affiliations:** a Faculty of Medicine and Health Science, Andrzej Frycz Modrzewski Krakow University, Krakow, Poland; b Department of Basic Biomedical Science, School of Pharmacy with the Division of Laboratory Medicine in Sosnowiec, The Medical University of Silesia in Katowice, Katowice, Poland

**Keywords:** Stress urinary incontinence, women’s health, medical students, medical education

## Abstract

**Background**: Stress urinary incontinence (SUI) is an embarrassing condition, which is one of the last taboos in modern medicine. The study aim was an attempt to assess medical students’ knowledge of female stress urinary incontinence.

**Methods**: The study involved 432 students of the Medical Department at the Medical University of Silesia in Katowice. Participants answered open-ended questions about: risk factors, prevention, diagnostic tests, conservative and surgical treatment in stress urinary incontinence.

**Results**: The obtained results indicated that female students know more about SUI than male students. Women – more often than men – could provide the definition of SUI (p < 0.01); additionally, they more frequently indicated prevention methods (p < 0.01), diagnostic testing (p < 0.001) and conservative methods of treatment (p < 0.001). Not all the respondents were able to properly define stress urinary incontinence. Risk factors were known to most of the respondents but only half of them were aware of surgical treatment and prevention methods. Even fewer answered questions about conservative treatment and diagnostic tests correctly.

**Conclusions**: We conclude that the knowledge of medical undergraduates who took part in the survey was not satisfactory. Most of the students were able to define properly the disease and point out risk factors. However, several steps should be taken to make stress urinary incontinence a disease much more known to medical students.

**Abbreviations**: SUI: Stress urinary incontinence; Group F: Females Group; Group M: Males Group; TVT: Tension Free Vaginal Tape; TOT: Transobturator Tape

## Background

Stress urinary incontinence (SUI) is one of the most important global health problems of the 21st century. It occurs when coughing, sneezing, laughing or hard physical labor increases pressure inside the abdomen, which is accompanied by the involuntary leakage of urine. Urinary incontinence worsens life quality in the occupational, social, mental, physical and sexual aspects of a woman’s life. Despite a real impact on the everyday lives of millions of women around the world, this problem is still disregarded and treated only as discomfort associated with personal hygiene [1–6].

Women accept their disability and do not seek medical help. This is a result of embarrassment, but also fear of going to the hospital and having possible surgery. With a lack of appropriate hygiene products or financial resources to purchase them, urinary incontinence may be a factor excluding a woman from normal life. Ignorance regarding the possibilities of conservative treatment causes women to deliberately give up their daily activities. Such behavior negatively affects social, family and matrimonial relations, and thus, drastically worsens the quality of a woman’s life, leaving her with a feeling of discomfort, helplessness, alienation and stress [1–4].

Women should know that involuntary urinary leakage while coughing, laughing or doing some physical activities is a symptom indicating a disorder in the functioning of the urogenital diaphragm. Moreover, women should be acquainted with exercises which strengthen the pelvic floor muscles, and know that they are the best method of preventing symptoms of urinary incontinence in the future [7–10]. Due to insufficient information about this disease and a small number of popular scientific publications, many people do not know about its existence, preventive measures and treatment. There are two reasons why women may not seek treatment for this condition. Firstly, due to a lack of knowledge, and secondly, because of embarrassment about raising the subject with their doctor. This can lead to a lot of suffering and unpleasant consequences which intensify with age [11].

Representatives of medical professions, especially doctors, should inform women about available conservative treatment and implementation as early as possible in order to allow such women to improve the quality of their everyday lives and return to normal social and family relationships. Doctors should skillfully overcome the patient’s embarrassment barrier, interview the patient and also inform them about the suspected health problem as well as inform them about the need to undertake diagnostics and treatment conducted by a doctor. Widening health education, including presenting preventive measures, is one of a doctor’s professional abilities. Medical personnel should inform their patients about how to modify their lifestyle to prevent stress urinary incontinence in a detailed manner.

Medical students were selected for the study because of their future contact with women at risk of or suffering from SUI. The authors wanted to check the knowledge of medical students who will become future doctors about stress urinary incontinence as in the near future, they will be primary care providers and the ones to be contacted in the case of various health problems, including those with urinary problems. Doctors are expected to know about SUI, especially as this is a common and concealed ailment. It is obvious that medical graduates will become further educated in various medical specialties, but still it does not exempt them from possessing knowledge on civilizational diseases. If a woman suffers from incontinence, she is likely to go to a gynecologist or urologist with this ailment; however if she chooses another specialist, they must also have some knowledge to talk about it with her, eliminate misconceptions, overcome the barrier of shame and, most importantly, provide her with prevention and conservative/surgical treatment options. It is difficult to undertake long-term preventive or therapeutic initiatives without a well-educated medical specialists who consider it necessary to act for the benefit of this specific issue. Available medical databases do not include any research on the knowledge of students who will become future medical personnel regarding stress urinary incontinence in women, which is one of the most hidden civilizational diseases, and one which has been passed over in silence. Thus, the aim of this study was to attempt to assess the knowledge of medical students about risk factors, prevention, diagnostic tests, and methods of conservative treatment used for stress urinary incontinence (SUI) in women.

## Methods

The study involved 282 women (group F – females) and 150 men (group M – males) of a mean age of 24.9 years (SD ± 0.96), who were students in their final year at the Medical Department of the Medical University of Silesia in Katowice. 6th-year students of the medical faculty were included in the study during the last semester of their studies, i.e., the 12th semester when they attended mandatory classes on the day of the survey. The survey was conducted at the courtesy of teachers who gave lectures on that day and allowed the questionnaire to be completed. Students who were absent from classes on the day of the survey were excluded from the questionnaire. Absent students would have been very likely to receive information from their fellow students about the SUI survey conducted at the University and could prepare their answers beforehand, which would in fact falsify the results. The study was based on the estimation of factual knowledge which doctors-to-be have, therefore the respondents were not informed that the survey is planned at the University. Before the surveys were distributed, the students were asked if they agreed to fill in a survey. The students were also informed that anyone refusing to fill in the survey should notify the person distributing the surveys of their choice. All the students in the study gave their consent to the survey and all accepted the survey questionnaires from the person distributing them and then returned them to the person who collected them afterwards.

The authors knew that these students had obligatory classes under their medical curriculum during which they were taught about incontinence issues. However, in order to further verify whether this was the case, the survey included the question about classes during which SUI was discussed. The question was: ‘what subjects during the course of your studies dealt with the problem of adult women’s stress urinary incontinence?’ The surveyed students enumerated many various classes during which SUI was discussed. These subjects were put in order and divided into categories. 1. Clinical treatment classes – this option was given by most, i.e., 86.1% of the students (these subjects were as follows: gynecology and obstetrics, surgery, urology, oncology), 2. Non-surgical clinical classes – this category was stated by 11.3% of the respondents (they included: internal diseases, clinical pharmacology, geriatrics, family medicine, nephrology, neurology, oncology, palliative care, pediatrics, medical propedeutics, sexology), 3. Classes in medical theory – such answers were given by 1.7% of the students (they were as follows: anatomy, medical biology, physiology, genetics, pathophysiology), 4. Diagnostic subjects – 0.5% of the respondents indicated such classes (they covered: diagnostic examinations, laboratory diagnostics, clinical biochemistry and pathobiochemistry). 5. Subjects in the field of prevention and social medicine – such subjects were indicated by 0.4% of the future doctors (they were: health prevention, prevention of women’s diseases, health promotion, psychology, sociology, public health, sexual and reproductive health, women’s health).

The study was based on an open-ended questionnaire assessing the knowledge of risk factors, prevention methods, diagnostic tests, as well as methods of conservative and surgical treatment used for SUI in women. Participants also had to properly define SUI by choosing one of four terms in each of three columns. The first column concerned urination (voluntary, involuntary, unexpected, planned). The second column asked about the amount of effort which is necessary for symptoms of urinary incontinence to occur (atypical, normal, high-performance, significant). The third column concerned situational incontinence (normal daily activities and behaviors, sport, neurological disorders, excessive hard work). Finally, the respondents had to indicate which medical professionals patients with SUI should see.

Excel 2016 and Statistica 9.0 software were used for archiving and statistical analysis. The chi^2^ test was used for analysis. A result of α = 0.05 was considered statistically significant.

## Results

**Figure 1.**
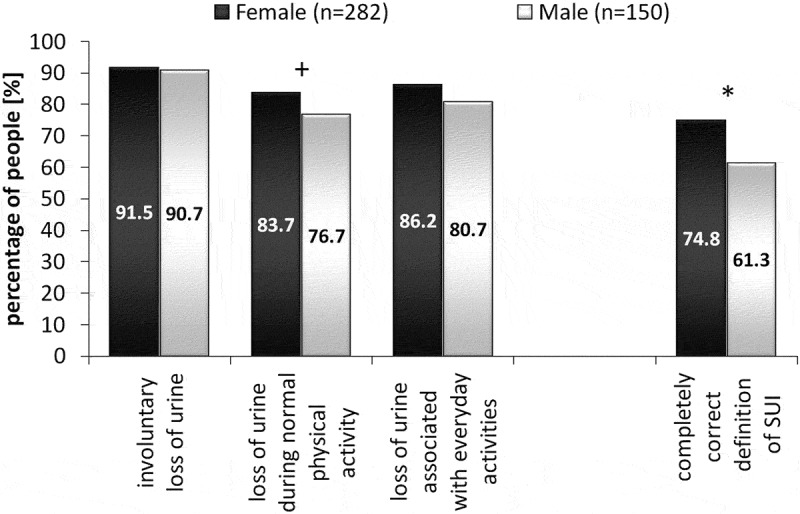
The percentage of male and female students who properly defined stress urinary incontinence (+p = 0.07, *p < 0.01).

In analyzing the individual elements of the question which defined SUI, it was noted that over 90% of the respondents in both groups correctly recognized urinary incontinence as the ‘involuntary loss of urine’ (Figure 1). 83.7% of the female group (group F) and 76.7% of the male group (group M) correctly pointed out that physical activity considered as ‘normal’ was enough to cause involuntary urination. 86.2% of group F and 80.7% of group M correctly believed that the effort associated with everyday activities could cause symptoms of SUI. However, the correct definition of SUI, which included all three elements, was given by 74.8% of female and 61.3% of male respondents. Therefore, there was a statistically significant difference between the two groups (p < 0.01) (Figure 1).

**Figure 2.**
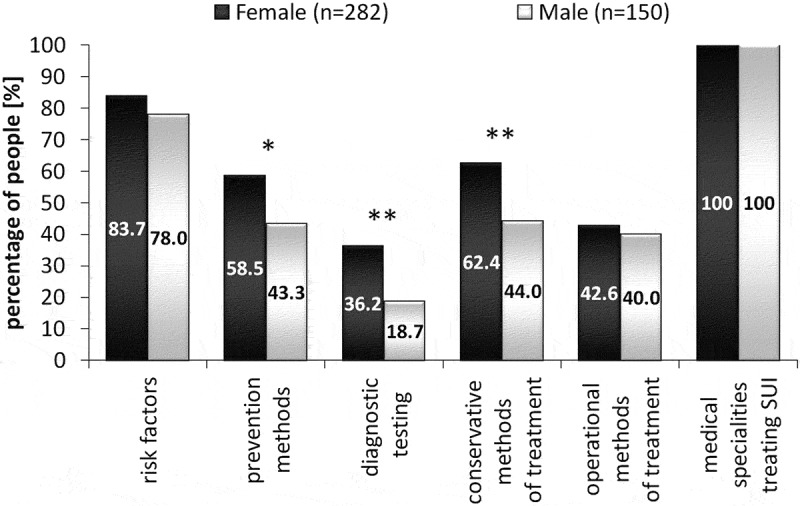
The percentage of male and female students declaring knowledge of risk factors, prevention methods, diagnostic testing, conservative and operational methods of treatment, and type of specialists who treat stress urinary incontinence (*p < 0.01, **p < 0.001).

Knowledge of conservative treatment methods was expressed by 62.4% of female and 44% of male respondents (Figure 2). Analysis showed statistically significant differences between the groups (p < 0.001). Similarly, more women than men showed knowledge of diagnostic tests for SUI in women. In the female group, the percentage was 36.2%, and in the group of male students only 18.7% (p < 0.001). SUI prevention methods were expressed by 58.5% of women and 43.3% of men. Most of the prevention methods were listed correctly. However, participants also suggested pharmacotherapy, which cannot be counted as a prevention method. Statistical analysis showed considerable differences between the groups of women and men (p < 0.01) (Figure 2). Risk factors for SUI in women were mentioned in both groups by similar numbers of students. The percentage of women was 83.7% and 78% among men (Figure 2). Similar numbers of students in both groups answered the question about methods of surgical treatment. The percentage was 42.6% among women and 40% among men. Most of the students mentioned methods of surgical treatment properly. Only a small percentage, i.e., 2.5% indicated the response ‘artificial sphincter’, which is not a surgical method used in female stress urinary incontinence. The groups of female students and male students of the medical department did not differ significantly in their knowledge of risk factors and methods of surgical treatment for SUI in women. Medical specialties helpful in treating this disease were given by all respondents (Figure 2).

**Figure 3.**
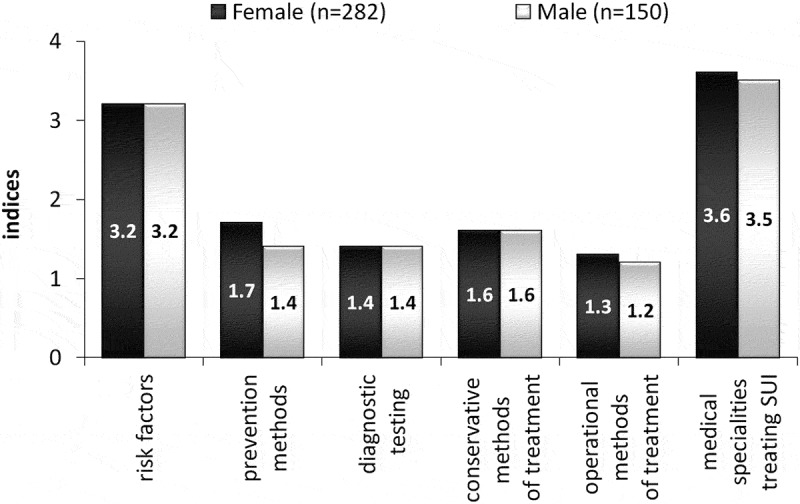
Indices for male and female students who mentioned risk factors, prevention methods, diagnostic testing, conservative and operational methods of treatment, and type of specialists who treat stress urinary incontinence.

Further analysis was only conducted on the responses of students who mentioned risk factors, prevention methods, diagnostic tests, methods of conservative and surgical treatment used in SUI, and medical specialties helpful in treating this disease. From the obtained results indices were calculated on the ratio of responses in each category to the number of people who responded in the group (Figure 3). The responding students listed more than three SUI risk factors and more than three medical fields treating SUI. The average number listed by the students: prevention methods, diagnostics testing, conservative and operational methods of treatment did not exceed two. The indices for risk factors, diagnostic tests, and methods of conservative treatment were identical in both groups. Small differences between the indices obtained in groups F and M related to methods of prevention, methods of surgical treatment, and medical specialization considered helpful in treating women suffering from SUI.

Most of the answers related to pregnancy and childbirth along with their respective complications (24.3%) were among the risk factors of SUI in women mentioned by the respondents (Table 1). The following less frequent responses were: age, menopause, obesity, surgery, urological diseases, genetic and developmental defects, and gynecological diseases. A wide range of other diseases and injuries such as: diabetes, hypertension, bronchial asthma, kidney disease, and urogenital injuries accounted for 10.8% of the enumerated risk factors. Moreover, SUI risk factors included: pelvic floor muscle weakness, hard physical effort and others. (Table 1). The most frequently mentioned among prevention methods were: pelvic floor muscle exercises (38.3% of responses, Table 1), and further: healthy lifestyle, physical activity, perinatal prophylaxis, and frequent health checks. Diagnostic tests mentioned by the respondents were the following: pad test, exercise test, coughing test and Q-tip test (40.7% of responses) along with urodynamic tests (37.9%).

**Table 1.** The percentage of risk factors, types of prevention, and diagnostic tests in stress urinary incontinence listed by medical students (n = number of responses in this category).

Risk factors (n = 1126)*	%	Types of prevention (n = 366)**	%	Diagnostic tests (n = 182)***	%
Pregnancy, childbirth, and their complications	24.3	Pelvic floor muscle exercises	38.3	Tests: pad, exercise, coughing and Q-tip	40.7
Age and menopause	17.4	Healthy lifestyle	22.4	Urodynamic tests	37.9
Obesity	13.1	Physical activity	12	USG, EMG, RTG	9.3
Surgery in the pelvic area	7.4	Perinatal prophylaxis	9.6	Gynecological and/or urological examination	5.5
Urological diseases	6.9	Frequent health checks	9	Others	6.6
Genetic and developmental defects	5.2	Avoiding excessive exercise	3.3		
Gynecological diseases	4.1	Medicines	3		
Other diseases and injuries	10.8	Educating women about stress urinary incontinence	2.2		
Pelvic floor muscle weakness	3.1				
Hard physical effort	2.6				
Others	5.1				

Number of respondents (percentage of respondents) *353 (81.7%); **230 (53.2%); ***130 (30.1%)

The methods of conservative treatment which the respondents suggested partly coincided with the previously mentioned prophylactic methods used in SUI in women. This particularly applied to answers regarding pelvic floor muscle exercises (39.1% of responses) (Table 2). Drugs (16.3%), general development exercises (15.5%) and physical therapy (5.4%) were less frequent responses. Avoiding cystitis, excessive exercise, obesity prevention, and regular urination were among other methods recognized by the students as conservative treatment (19.9% of responses). Surgical methods mentioned most often by the students were: sling procedures (67.5%, Table 2), vaginal suspension was mentioned less often and an artificial sphincter was indicated very rarely (Table 2). The respondents suggested specialists which a woman should see when suffering from SUI. They mainly included: gynecologist, urologist and family doctor, followed by neurologist and geriatrician (Table 2). It should, however, be noted that 7.4% of the responses given by the medical students when selecting a specialist that a woman experiencing problems with incontinence stated: nephrologist.

**Table 2.** The percentage of methods of conservative and surgical treatment used in stress urinary incontinence, and types of specialist who treat urinary incontinence indicated by medical students (n = number of responses in this category).

Methods of conservative treatment (n = 386)*	%	Methods of surgical treatment (n = 216)**	%	Methods of conservative treatment (n = 386)*	%
Pelvic floor muscle exercises	39.1	Sling procedures	67.5	Pelvic floor muscle exercises	39.1
Drugs	16.3	Vaginal suspension	30	Drugs	16.3
General development exercises	15.5	Artificial sphincter	2.5	General development exercises	15.5
Physical therapy	5.4			Physical therapy	5.4
Visiting a specialist for check-ups	2.1			Visiting a specialist for check-ups	2.1
Vaginal balls or cones	1.6			Vaginal balls or cones	1.6
Others	19.9			Others	19.9

Number of respondents (percentage of respondents) *242 (56.0%); **180 (41.7%); ***432 (100%)

## Discussion

Health is not permanent, immutable and inviolable. It can be assisted by appropriate pro-health measures. However, one’s health may be damaged in both direct – through improper actions – as well as in an indirect manner – by long-term neglect. No doubt the basis for public interest in health-related behavior is the widespread implementation of health education. Such an education should include not only sharing knowledge, but also developing appropriate health behaviors and attitudes towards risk factors, which could change the way people think about health. In other words, healthcare professionals should develop a pattern of ‘individual health-related lifestyles’ shaped by conscious choices. This is often difficult because the range of possibilities open to a patient depends on their socio-economic conditions. Therefore, patients should receive all the necessary information on prevention methods from their doctors, especially general practitioners. In particular, so-called ‘civilizational diseases’, or those which affect people of a certain age, need to be brought to the attention of patients. Between visits to a specialist, patients should have control over their own bodies and health.

The study shows that students in the final year of medical studies had problems with the correct definition of stress urinary incontinence. The correct definition was only provided by about 75% of women and 61.3% of men. This percentage should be considered unsatisfactory, especially in the case of the male students, because they had a choice of options offered in the survey and only had to choose the correct answer.

The risk factors for stress urinary incontinence in women mentioned by the students were largely consistent with those reported in literature. They especially concerned the impact on the occurrence of SUI symptoms of menopause, obesity, pregnancy and childbirth [8–14]. However, 16% of women and 22% of men did not answer this question.

Knowledge of prevention methods was expressed by 59% of women and 43% of men. This is a low percentage given the fact that the answers were provided by people who are to become doctors in the near future. If they meet women suffering from SUI, they should know how to handle the matter. Mentioning the known methods used in preventing SUI, students concentrated on pelvic floor exercises, healthy lifestyle, moderate physical activity, obesity prevention and avoiding excessive effort. The students also pointed out the need for perinatal prevention, which is one of the major factors preventing SUI. A small number of the respondents identified the need for educating women about SUI. Lectures, presentations and leaflets needed for disseminating knowledge should be addressed to both young women and those in menopause. Such action is necessary because many women are still unaware of having this problem or risk of the disease, and that it is a condition that can and should be treated [15–19].

A similarly low proportion of respondents gave answers regarding conservative medical treatment. The responses were similar to previously reported methods for prevention. They particularly coincided with pelvic floor muscles exercises. Most of the literature data reports that conservative treatment should, in particular, include exercise of the pelvis minor muscles. Such methods of treatment are considered safe, since there have been no complications or adverse side effects of therapy. These exercises can be performed in a variety of forms: as free exercises, combined with biofeedback, as well as exercises performed with the help of balls or vaginal cones. Apart from these exercises, physiotherapy as conservative treatment for stress urinary incontinence uses electrotherapy, particularly percutaneous pelvic floor electrical stimulation and vaginal electrical stimulation, magnetic therapy, and laser therapy [20–22]. Some students suggested visiting a specialist for check-ups as a conservative treatment method. This should be considered as a mental shortcut. A visit to a doctor cannot be regarded as a means or a method of treatment for any medical condition.

The responses of the students were quite short and vague on medicine as a method of conservative treatment. In female stress urinary incontinence, medications which include estrogen are applied. The use of exogenous estrogen causes: an increase in urethral closure pressure due to increased blood flow through the submucosal vascular plexus, activation of collagen gene expression, stimulation of fibrillar collagen production (a component of the filamentary pubourethral fascia), increased the threshold of sensitivity of the bladder to stretching, and a reduction of recurrent urinary tract infections by restoration of the normal vaginal ecosystem [23–25].

A surprisingly low percentage of the medical students, especially men (19%), displayed knowledge of the diagnostic methods in SUI. In addition, twenty students (5.5% of responses) thought a gynecological and/or urological examination was a diagnostic method. The lack of sufficient knowledge on the subject among our young doctors indicates that they cannot take effective action and help patients suffering from SUI.

The ineffectiveness of conservative treatment and the often considerable severity of the disease caused by no previous treatment necessitate surgical intervention. The highest percentage of responses concerned tapes: TVT (*tension- free vaginal tape*) and TOT (*transobturator tape*). Operations with TVT tapes are widely described in the literature because they are simple to perform. They have a high clinical effectiveness and a low percentage of intraoperative and post-operative complications [26,27].

SUI is a chronic disease that has a huge impact on a patient’s quality of life and on their social and professional status [1–4]. Therefore, it is necessary to draw the attention of medical authorities to this problem, and to raise their awareness of this subject in order to encourage them to widen knowledge that will make it possible to take appropriate action for the benefit of women suffering from urinary incontinence. The task of medical personnel is not only to promote knowledge about urinary incontinence among women of all ages, but also to eliminate misconceptions and the taboo surrounding it. Women should be motivated to seek help to be provided with assistance to understand the disease, and access to new diagnostic and treatment methods should also be increased. During the first interview with a patient general practitioners should cross the embarrassment barrier and ask the patient if they have been experiencing any symptoms of this disorder, and if so, initiate appropriate treatment. Questions about urinary incontinence should be routinely asked to: multiparous women, women suffering from chronic constipation, chronic inflammation of the respiratory tract with a chronic cough, obese women and menopausal women. Unfortunately, in most cases doctors do not ask their patients about urinary incontinence, because they do not want to make them feel uncomfortable, and the patients do not mention their embarrassing symptoms. For the doctor, it should be the symptom that makes it possible to recognize the pathology and, subsequently, implement an individual therapeutic process for the patient. In this situation, only appropriate education, awareness of the problem and great tact and sensitivity will make it possible to help the patient cross the embarrassment barrier and engage in a sincere conversation about this condition.

**Figure 4.**
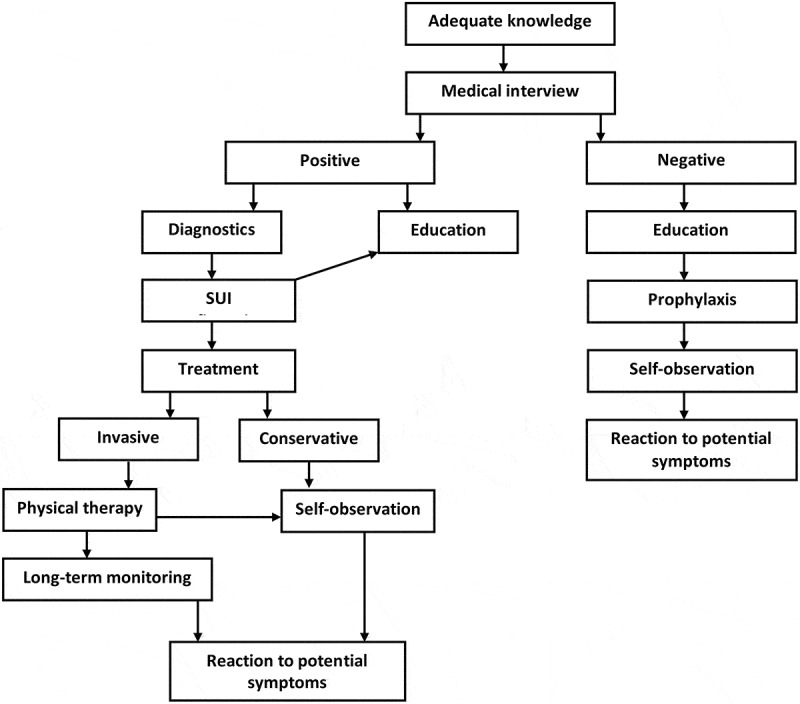
Suggested model of preventive and therapeutic measures for medical staff in contact with women at risk and/or suffering from stress urinary incontinence.

Under the final analysis of this issue, the authors of this paper suggested a model of preventive and therapeutic measures which may help both medical staff and women at risk and suffering from stress urinary incontinence to take appropriate and structured measures to prevent SUI (Figure 4). Healthcare professionals should consider a model of preventive and therapeutic measures for women with SUI which should be accepted and disseminated (Figure 4). The first and fundamental step towards treating women suffering from urinary incontinence problems is that medical personnel and, above all, doctors should have adequate knowledge of this ailment. Such knowledge will let them to conduct a medical interview in order to specify further stages of therapeutic treatment. If a doctor finds no symptoms or suspicions of stress urinary incontinence (SUI negative), they should take basic educational measures to bring a patient closer to the issue of SUI. This education should include, among others, the presentation of potential solutions offered by prophylaxis, which helps to avoid or reduce its troublesome symptoms in the future, recommendation of self-observation and early reaction to alarming symptoms. However, if there is any suspicion that a woman may be suffering from stress urinary incontinence (SUI positive), immediate diagnostics should be undertaken. This woman should see a specialist doctor to recognize the symptoms and their causes. It is also important to simultaneously undertake educational measures towards this woman in the range of this disease, including potential diagnosis, prevention and treating of this ailment. The diagnosis of urinary incontinence should be based especially on a urodynamic examination, which allows a final diagnosis to be made. If the doctor does not confirm the initial diagnosis of incontinence, the only and the best way to follow is to re-educate such a patient on the illness and its symptoms which may be considered alarming. If the doctor’s suspicions are objectively confirmed, the patient is provided with treatment options, explaining the causes, course and prognosis of this disease at the same time. Therapeutic measures may include: conservative treatment, including pelvic floor muscle training conducted in various forms by physiotherapists, nurses or midwives, and self-observation for symptoms of this disease. The second possible therapeutic path refers to invasive treatment which is considered to be the competence of medical specialists. A surgical procedure, however, is not the end of the presented path, as even afterwards a given woman must undertake long-term self-monitoring and reaction to potential symptoms and still expand her knowledge about the objectives and options of physical therapy in preventing the relapse of this disease. More often than not, by the time a doctor conducts an interview with a patient, the disease is already in an advanced state. To avoid this and help women realize that an early diagnosis gives a better chance of being cured or easing symptoms, large-scale educational efforts should be made (brochures, leaflets, articles in popular science literature and women’s magazines). Representatives of all health professions, especially doctors, ought to be encouraged to take action and overcome the embarrassment barrier in patients. They should talk to women who may be concealing their symptoms. Thanks to activities aimed at spreading health education about stress urinary incontinence, patients who so far have hidden their ailment through shame, may be convinced about the necessity of using medical advice and may become informed about possible treatment. Poor knowledge about therapeutic options among women and lack of available information materials at healthcare related facilities (such as clinics, pharmacies) demonstrate that the importance given to this disease is insufficient.

On the basis of our study we conclude that the knowledge of final year medical students about stress urinary incontinence in women is not satisfactory. It should also be noted that the female students showed greater knowledge than the male students, as confirmed by our statistical analyses several times. This may be due to the fact that female students are much more interested in issues related to women’s health. The problem of urinary incontinence is similarly common not only in Poland but also in Western Europe and the USA. We can assume that the situation presented in this paper, which is based on the Polish experience, is similar to the situation in other countries.

## Conclusions

Medical students did not show satisfactory knowledge of stress urinary incontinence in women. The percentage of students demonstrating their knowledge and providing detailed responses was not sufficient. However, the respondents who tried to answer the questions about risk factors, diagnostic tests, and methods used in the surgical treatment of this condition provided mainly correct responses. The results presented in this study point to many problems with raising the awareness of women suffering from stress urinary incontinence. They indicate an urgent need to develop and implement educational programs for medical students which include the issue of SUI.

## Data Availability

The datasets used and/or analyzed during the current study are available from the corresponding author upon reasonable request.
